# Amputation during Anæsthesia with Chloral

**Published:** 1870-09

**Authors:** 


### Amputation during Anæsthesia with Chloral.

Dr. Noir (of Briude, France,) has published in the *Gazette des Hopitaux* of December 23, 1869, the case of a man aged sixty-four, suffering acutely from osteosarcoma of the leg. The patient was very anxious to have the limb taken off; and, as a trial, he took about sixty grains of chloral, dissolved in two ounces of simple syrup, at 8 A. M. Up to nine o'clock, he frequently made efforts at vomiting, and had defective vision; after this came violent excitement, which lasted two hours; he then fell asleep, and soon was so insensible that he could be moved about without waking. This sleep lasted about an hour and a half, and the patient, on coming to his senses, said he felt very well, and asked for food. Pain had of late deprived him of sleep, and he was overjoyed to have had some rest.

Two days after this, the man took seventy-five grains of chloral at eight in the morning, and was uncomfortable for two hours, when he fell into a deep slumber, and underwent amputation of the leg without moving or uttering a sound. After being placed in bed, the patient sank into an alarming coma for one hour; after which, on waking, he was seized with violent delirium and severe vomiting. These fearful symptoms lasted about seven hours, when the poor man passed into a state of complete prostration, and recovered his senses; but did not recollect anything of what had passed, and could hardly speak or move. He took some beef-tea, had a sleepless but quiet night, and the next day all the effects of the chloral had passed off.

Dr. Noir remarks that, in this case, delirium, prostration, and coma, were so alarming that it would be imprudent to use chloral as an anæsthetic in operations if further experience prove that these dangerous symptoms regularly present themselves. There can however, be no doubt, he adds, that insensibility was complete during the operation.—*Lancet*, January 1, 1870.

